# Association between age at onset of independent walking and objectively measured sedentary behavior is mediated by moderate-to-vigorous physical activity in primary school children

**DOI:** 10.1371/journal.pone.0204030

**Published:** 2018-09-18

**Authors:** Tomoko Aoyama, Shigeho Tanaka, Maki Tanaka, Masayuki Okuda, Shigeru Inoue, Chiaki Tanaka

**Affiliations:** 1 Department of Nutritional Epidemiology and Shokuiku, National Institute of Health and Nutrition, National Institutes of Biomedical Innovation, Health and Nutrition, Shinjuku-ku, Tokyo, Japan; 2 Department of Nutrition and Metabolism, National Institute of Health and Nutrition, National Institutes of Biomedical Innovation, Health and Nutrition, Shinjuku-ku, Tokyo, Japan; 3 Department of Child Education, Kyoto Seibo College, Kyoto-shi, Kyoto, Japan; 4 Department of Environmental Medicine, Graduate School of Science and Engineering, Yamaguchi University, Ube-shi, Yamaguchi, Japan; 5 Department of Preventive Medicine and Public Health, Tokyo Medical University, Shinjuku-ku, Tokyo, Japan; 6 Division of Integrated Sciences, J. F. Oberlin University, Machida-shi, Tokyo, Japan; University of Sydney, AUSTRALIA

## Abstract

**Objective:**

Age at onset of walking has been shown as an early predictor of physical activity in infants and children. However, little is known about whether age at onset of walking may predict sedentary behavior (SB). The aim of the present study was to examine the association between the timing of onset of walking and objectively measured SB, and whether this association is mediated by moderate-to-vigorous physical activity (MVPA) in children.

**Methods:**

The subjects were 388 elementary school children aged 6–12 years. Current weight and height data were collected. Birth weight and the age in months the child first walked independently were reported based on the parents’ recall. Children’s SB and physical activity were objectively measured using a triaxial accelerometer (Active style Pro HJA-350IT, OMRON). The following summary outcome variables were derived from accelerometer data: Time (min/day) spent in SB (≤1.5 metabolic equivalents [METs]) and MVPA (≥3.0 METs).

**Results:**

The mean ± *SD* time (min/day) spent in sedentary was 376 ± 62 and MVPA was 67.6 ± 20.8. Multiple linear regression analyses revealed that a later age at independent walking was associated with increased time spent in SB (*β* = 0.15, *P* < 0.001) and decreased time spent in MVPA (*β* = -0. 18, *P* < 0.001) after adjusting for gender, birth weight, current age, body weight, schools, and time spent wearing the accelerometer. When MVPA was introduced as a covariate in the model predicting SB, the association between the age at independent walking and time spent in SB was completely attenuated (*β* = 0.04, *P* = 0.215), while MVPA was significantly associated with SB (*β* = -0.61, *P* < 0.001).

**Conclusions:**

Our results indicate that infants who walked at a later age spent more time in SB in childhood, and this association is mediated by MVPA. Appropriate interventions which focus on increasing MVPA and thereby reducing SB may be beneficial in infants who demonstrate a later age at onset of independent walking.

## Introduction

Numerous epidemiological studies have identified consistent associations between physical inactivity, defined as “not performing sufficient amounts of moderate-to-vigorous physical activity (MVPA)”[1], and all-cause and cardiovascular disease mortality [2,3].

Sedentary behavior (SB), defined as “any waking activity characterized by an energy expenditure ≤1.5 metabolic equivalents and a sitting or reclining posture” [4], has also increasingly been recognized as an important risk factor associated with health outcomes [5]. Recent studies have shown that having a high level of SB negatively impacts health after considering the benefits of MVPA [6]. SB is an independent risk factor for diseases and is therefore worth considering in addition to MVPA. Sedentary lifestyles have become major health problem worldwide, and the identification of factors which determine SB is of great importance to public health.

Previous reviews have shown that SBs [7] and physical activities [8] track at moderate levels from childhood, suggesting that SB and physical activity (PA) during childhood may have a particularly important role over an individual’s life course. Thus, a better understanding of the determinants of children’s SB and PA is needed in order to effectively decrease SB and increase PA across the lifespan. Systematic reviews have mainly focused on environmental [9], psychological, social, and behavioral [10,11] factors as correlates of SB and PA during childhood and adolescence. Recent evidence also suggests that early-life factors make a significant contribution to SB [12] and PA [13] in young people, and a few studies have shown that indicators of motor development were an early predictor of PA in children [14–16].

A birth cohort study by Ridgway et al. (2009) reported that an earlier age at standing unaided and walking supported in infancy predicts higher levels of PA as indicated by an increased frequency of sports participation during adolescence [14]. Mattocks et al. (2008) reported motor coordination at 6 months, which was based on combined score from 12 questions, was positively associated with objectively measured PA (cpm) using accelerometry in children aged 11–12 years [15]. Associations between age at onset of walking and objectively measured PA patterns already exist in the first two years [17,18]. Hnatiuk et al. (2013) reported that 19-month-old toddlers who walked earlier had a higher total time spent in light-to-vigorous-intensity PA than those who walked later, regardless of how long the toddler had been walking [17]. A study by Prioreschi et al. (2017) reported diurnal distributions of mean vector magnitude by developmental stage and showed that in most cases walkers were more active during the day than crawlers, even after adjusting for age [18]. Therefore, these associations between age at onset of walking and PA during the first two years may carry over to childhood.

We also recently reported that a later age at onset of independent walking predicts lower levels of MVPA (min/day) measured by an accelerometer in 6- to 12-year-old children [16]. However, no studies have shown significant associations between indicators of motor development and SB. It’s important that increased SB and decreased MVPA often occur in combination, although these factors are mutually independent to some degree [19]. Therefore, it is plausible that a later age at onset of walking may also predict increased time spent in SB. However, interventions designed to increase PA or MVPA generally resulted in reductions in sedentary time [20,21]. These studies suggest that the differences in sedentary time may be partly caused by the amount of MVPA in an individual. Therefore, we hypothesized that MVPA might mediate a potential association between age at independent walking and SB.

In order to develop successful strategies to prevent prolonged sedentary time during childhood, it is essential to elucidate the substantial role of the timing of onset of walking in the determining levels of SB in later life, taking MVPA into consideration. The aim of the present study was to examine the association between the age at onset of independent walking and objectively measured SB, and whether this association is mediated by MVPA in children.

## Materials and methods

### Study subject

The subjects were Japanese primary school children, who were recruited from 14 primary schools in urban areas of Tokyo, Kanagawa, and Kyoto prefectures. The anthropometry, accelerometry, and questionnaire data were collected from June 2012 to January 2015 during the school year.

A total of 569 individuals participated in this study. A questionnaire filled out by parents was used to evaluate the children’s medical history, bedtime and wake-up time, age at onset of independent walking, and birth weight. Those who rescinded their consent (n = 8), had no questionnaire data (n = 16), or had a history of conditions affecting PA such as respiratory disease or heart disease (n = 28) were excluded. Additional subjects were excluded if the accelerometer data did not conform with the study criteria (see below) (n = 91). A final data set for 388 children was used for subsequent analyses after the exclusion of children with incomplete data on age at onset of independent walking (n = 24) including an outlier (≥25 months) [22], those born with a very low birth weight (<1.5 kg) (n = 2), and those who were multiples (n = 12).

The research project was approved by the Ethical Committee of Oberlin University (receipt number: 12023). The study procedures were explained in writing to all children and parents, and written informed consent was obtained from each participant and his/her parents.

### Age at independent walking and birth weight

Information on independent walking and birth weight in children was retrospectively reported on a questionnaire according to the parents’ recall. The parents were asked to provide their children’s birth weight and the age in months when their children were first able to walk without assistance [23,24]. Birth weight was investigated as a factor potentially related to SB [25] and PA [13].

### Sedentary behavior and physical activity

Daily SB and PA were objectively measured using a triaxial accelerometer (Active style Pro HJA-350IT, Omron Healthcare, Kyoto, Japan; dimensions 74 × 46 × 34 mm and weight 60 g including batteries) for seven consecutive days. The device is described in detail elsewhere [26]. The subjects wore the accelerometer on the left side of the waist and were requested to wear the device at all times except under special circumstances such as dressing, bathing, and swimming. An epoch of 10 seconds was used, and the data were converted using following conversion equations for primary school children based on the results of Hikihara et al. (2014), because the metabolic equivalent (MET) values recorded by the accelerometer are overestimated in primary school children [26].

Ambulatoryactivities:0.6237×METvalueofActivestylePro+0.2411

Non‑ambulatoryactivities:0.6145×METvalueofActivestylePro+0.5573

Ambulatory activities (e.g., walking and running) and non-locomotive activities (e.g., playing games, cleaning, playing with blocks, tossing a ball, and aerobic dance) were discriminated based on the ratio of unfiltered synthetic acceleration to filtered synthetic acceleration [26]. Filtered synthetic acceleration was defined as the integrated acceleration ((X^2^+ Y^2^+ Z^2^)^0.5^) after the gravitational acceleration was removed from each dimensional acceleration (X, Y, Z) by passing it through a second-order Butterworth high-pass filter with a cut-off frequency of 0.7 Hz [27].

We analyzed data collected between 7:00 and 21:00 to exclude sleep. The average (± SD) bedtime and wake-up time of children assessed by a questionnaire were 21:27 ± 2:00 and 6:49 ± 0:27, respectively, in this study. Therefore, we determined the time window for the analyses as above (between 7:00 and 21:00). Awake data were partly excluded but data accumulated during sleep were not included in the analyses in many cases. It was difficult to discriminate the bedtime and wake-up time of each child day by day. If sleep periods were included in the time window (7:00 to 21:00), misclassifications of sleep into SB would increase, while excluding awake periods between 21:00 and 7:00 from the analyses would hardly affect duration of MVPA because little MVPA is observed during the time immediately after wake-up and before bedtime. Subjects with data obtained from wearing the accelerometer >10 hours on at least two weekdays and at least one weekend day [28,29] were included in the analysis. Periods with >60 min of consecutive zero counts (no signal) were classified as “no wearing time”.

Three variables were analyzed in this study: time (min/day) spent in SB (≤1.5 METs), light PA (LPA, 1.6–2.9 METs), and MVPA (≥3.0 METs). The mean weekly values were then calculated. The mean values were calculated by weighting for five weekdays and two weekend days (weighted data = ([mean for weekdays × 5] + [mean for weekend days × 2]) / 7).

### Anthropometry

Body height and weight were measured without shoes, but with clothing to the nearest 0.1 cm and 0.1 kg, respectively. We used scales that were typically used in primary schools; otherwise, we brought a “Karada Scan HBF-370” (Omron Healthcare, Kyoto, Japan) scale into the schools and used it. Net body weight was calculated as the measured body weight minus the weight of the clothing. We used 0.5 kg as the weight of clothing in all children except for children who underwent measurements in physical exercise uniforms; their light clothing was regarded as weighing 0.35 kg. The weight used for clothing was determined by weighing typical children’s clothing.

### Statistical analyses

The measured and calculated values are presented as means ± standard deviations (*SD*). Student’s *t*-test was used to investigate potential differences between boys and girls. Partial correlation analysis was used to test the relationships between study variables controlled for gender and months of age as covariates. Multiple linear regression analyses were performed to assess the associations between age at onset of independent walking and SB or PA. We first entered the age at independent walking (Model 1) and then added the birth weight and current weight as independent variables to examine how the association is altered by body weights (Model 2). In order to investigate whether MVPA acts as a mediator in the association between motor development and SB, we finally introduced MVPA as a covariate in the model for predicting SB according to the methods suggested by Baron and Kenny [30].

For illustrative purposes, the age at onset of walking was categorized into three groups according to tertiles: early (10.4 ± 0.7 months), middle (12.4 ± 0.5 months), and late (15.4 ± 2.0 months). Linear trends of SB, LPA, and MVPA were evaluated using an ordinal variable for the three groups [31]. All models were adjusted for gender, birth weight, current weight, months of age, interaction term (gender × months of age), schools, and accelerometer wearing time.

The statistical analyses were performed using IBM SPSS statistics 22.0 for Windows (IBM Japan Ltd., Tokyo, Japan). The statistical significance level was set at *P* < 0.05.

## Results

The characteristics of the subjects are shown in Table 1. The average age of the subjects was 111.8 ± 19.4 months for boys and 112.0 ± 19.0 months for girls. No significant differences between boys and girls were observed in current age, height, weight, or the age at which they walked independently in infancy. Birth weight was slightly higher in boys than girls (95% CI: 7.5, 161.2). Boys showed higher MVPA values compared with girls (95% CI: 13.3, 20.9), whereas SB was significantly lower in boys than in girls (95% CI: -26.3, -1.6).

**Table 1:** Characteristics of the 388 children aged 6–12 years.

Variables	All (n = 388)		Boys (n = 179)	Girls (n = 209)	*P* value^†^
Months of age (mos.)	111.9 ± 19.2	(73–151)	111.8 ± 19.4	112.0 ± 19.0	0.929
Height (cm)	132.5 ± 10.7	(107.5–163.5)	132.4 ± 10.0	132.6 ± 11.3	0.804
Weight (kg)	29.2 ± 7.6	(16.4–57.2)	29.7 ± 7.9	28.7 ± 7.2	0.178
Birth weight (g)	3045 ± 385	(1678–4288)	3090 ± 376	3006 ± 390	0.032
Age at independent walking (mos.)	12.8 ± 2.3	(8–24)	12.9 ± 2.4	12.8 ± 2.2	0.414
8 mos. (%)	0.5		0.6	0.5	
9 mos. (%)	2.6		2.2	2.9	
10 mos. (%)	8.2		8.4	8.1	
11 mos. (%)	15.5		14.5	16.3	
12 mos. (%)	25.8		26.8	24.9	
13 mos. (%)	14.7		12.8	16.3	
14 mos. (%)	13.7		13.4	13.9	
15 mos. (%)	10.1		10.1	10.0	
16 mos. (%)	2.8		3.4	2.4	
17 mos. (%)	2.1		3.4	1.0	
18 mos. (%)	1.8		2.2	1.4	
≥19 mos. (%)	2.3		2.2	2.4	
SB (min/day)	376 ± 62	(204–550)	368 ± 59	382 ± 64	**0.027**
LPA (min/day)	359 ± 50	(219–516)	354 ± 48	363 ± 51	0.060
MVPA (min/day)	67.6 ± 20.8	(17.6–134.5)	76.8 ± 20.0	59.7 ± 18.1	**<0.001**

Data are means ± *SD* (range) or proportions.

SB, sedentary behavior; LPA, light physical activity; MVPA, moderate-to-vigorous physical activity.

^†^ *P* values were calculated for gender difference by *t*-test.

Table 2 shows the partial correlation matrix between early life factors (birth weight and age at onset of independent walking) and current measures (height, weight, SB, LPA, and MVPA) controlled for gender and months of age as covariates. Birth weight was positively and weakly correlated with current height (*P* < 0.01) and weight (*P* < 0.05). The age at independent walking was weakly correlated positively with current weight (*P* < 0.05) and SB (*P* < 0.01) and inversely with MVPA (*P* < 0.001), but these associations were weak. Current height (*P* < 0.05) and weight (*P* < 0.01) were also weakly associated with SB and LPA. SB was strongly associated with LPA and moderately associated with MVPA (*P* < 0.001). LPA was weakly associated with MVPA (*P* < 0.001).

**Table 2:** Partial correlation matrix controlled for gender and months of age.

Variables	Age at independent walking (mos.)	Height (cm)	Weight (kg)	SB (min/day)	LPA (min/day)	MVPA (min/day)
Birth weight (g)	-0.030	0.148**	0.125*	-0.039	0.012	0.052
Age at independent walking (mos.)		-0.077	-0.111*	0.149**	-0.074	-0.200***
Height (cm)			0.722***	0.123*	-0.125*	-0.055
Weight (kg)				0.158**	-0.150**	-0.073
SB (min/day)					-0.731***	-0.575***
LPA (min/day)						0.352***

* *P* < 0.05.

** *P* < 0.01.

*** *P* < 0.001.

SB, sedentary behavior; LPA, light physical activity; MVPA, moderate-to-vigorous physical activity.

**Fig 1:**
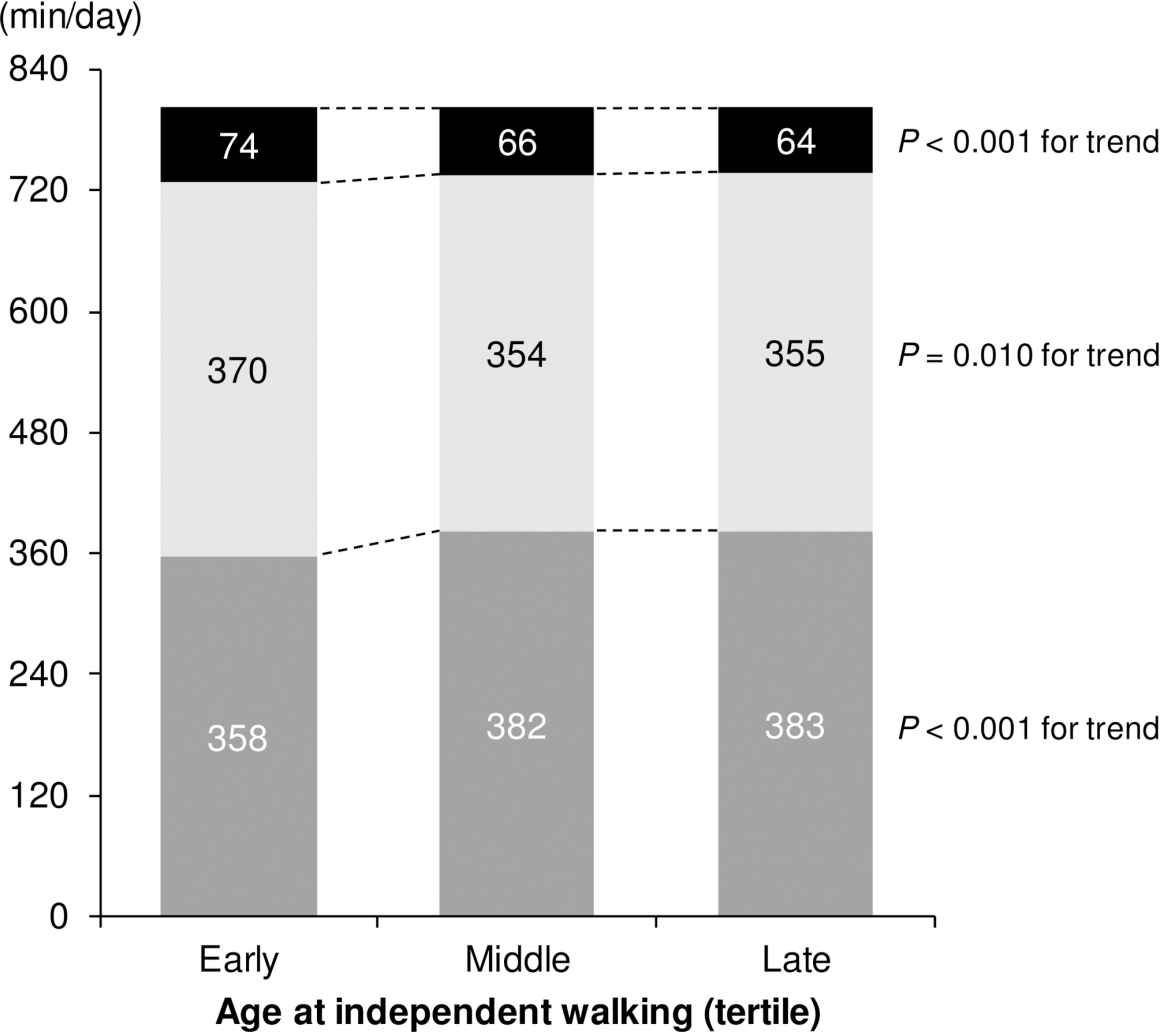
Estimated means of SB and PA among tertiles of the age at independent walking. (Dark gray) SB. (Light gray) LPA. (Black) MVPA. Adjusted for gender, months of age, birth weight, current weight, interaction (gender × months of age), schools, and accelerometer wear time. SB, sedentary behavior; LPA, light physical activity; MVPA, moderate-to-vigorous physical activity.

Table 3 presents the results of the multiple linear regression analyses used to examine the associations of age at independent walking with SB and PA. As shown in model 1, the age at independent walking was positively associated with SB (*β* = 0.13, 95% CI: 4.40, 17.37) and inversely associated with MVPA (*β* = -0.17, 95% CI: -6.72, -2.35) after adjusting for gender, months of age, interaction term (gender × months of age), schools, and accelerometer wear time. A further adjustment for birth weight and current weight made little difference in the relationships between age at independent walking and SB (*β* = 0.15, 95% CI: 5.43, 18.30) and MVPA (*β* = -0.18, 95% CI: -6.93, -2.56) (model 2 and Fig 1).

**Table 3:** Multiple linear regression analyses with SB or PA as the dependent variable.

Independent variables	SB (min/day)			LPA (min/day)			MVPA (min/day)		
	*B*	*β*	*P*	*B*	*β*	*P*	*B*	*β*	*P*
**Model 1**									
Age at independent walking (mos.)	10.88	0.13	**0.001**	-6.35	-0.10	**0.022**	-4.54	-0.17	**<0.001**
**Model 2**									
Age at independent walking (mos.)	11.86	0.15	**<0.001**	-7.12	-0.11	**0.010**	-4.74	-0.18	**<0.001**

**Model 1**: Adjusted for gender, months of age, interaction (gender × months of age), schools, and accelerometer wear time.

**Model 2**: As Model 1 plus birth weight and current weight.

*B*, unstandardized regression coefficient; *β*, standardized regression coefficient.

SB, sedentary behavior; LPA, light physical activity; MVPA, moderate-to-vigorous physical activity.

When MVPA was introduced as a covariate in the model predicting SB (Table 4), the association with the age at independent walking was completely attenuated and ceased to be significant (*β* = 0.04, 95% CI: -1.92, 8.49) and MVPA was significantly associated with SB (*β* = 0.61, 95% CI: -2.05, -1.57).

**Table 4:** Multiple linear regression analysis with SB as the dependent variable.

Independent variables	SB (min/day)		
	*B*	*β*	*P*
Age at independent walking (mos.)	3.29	0.04	0.215
MVPA (min/day)	-1.81	-0.61	**<0.001**

Adjusted for gender, months of age, birth weight, current weight, interaction (gender × months of age), schools, and accelerometer wear time.

*B*, unstandardized regression coefficient; *β*, standardized regression coefficient.

SB, sedentary behavior; MVPA, moderate-to-vigorous physical activity.

We also examined the associations between age at independent walking and SB, LPA, and MVPA in boys and girls separately (S1–S3 Tables). As a result, similar results were obtained in both genders, except that the association between the age at independent walking and LPA was not significant in boys (*β* = -0.07, *P* = 0.335 for model 1; *β* = -0.09, *P* = 0.199 for model 2), while the association was still significant in girls (*β* = -0.12, *P* = 0.029 for model 1; *β* = -0.13, *P* = 0.024 for model 2) (S2 Table).

## Discussion

This study was performed to examine the association between age at onset of independent walking and SB in children and to test whether this association is mediated by MVPA. The main finding of this study was that later age at independent walking was significantly (albeit weakly) associated with increased time spent in SB. To our knowledge, only one study by Wijtzes et al. (2013) has investigated the association between motor development and SB, and the investigators found no association between having a delayed gross motor development at one year and sedentary time in two-year-old toddlers [32]. As such, we believe that the present study is the first to show the link between an indicator of infant motor development and objectively measured SB. The difference in accelerometry may be a potential reason for the discrepancy between our findings and those of Wijtzes et al. (2013); They used a uniaxial accelerometer (ActiGraph) during one weekday and one weekend day in their study [32], while we used a triaxial accelerometer during seven days. The mean wearing days of accelerometer in our study (6.3 ± 1.0 days) was considerably greater than the minimum criteria (at least three days) which is required for reliable PA monitoring in young children [28,29]. Our results indicate that on average, each increase of one month in a child’s age at independent walking was associated with almost 12 min/day more of SB (Table 3, model 2). This may suggest important clinical implications, given that the age at which children first walk independently is distributed over more than a nine-month range [22].

However, an important possibility to be considered was that MVPA may play a mediator role in the observed association between age at independent walking and SB in model 2. To elucidate the substantial association between the timing of onset of independent walking and SB, we tested whether this association is mediated by MVPA. As a result, it was found that the association between the age at independent walking and the time spent in SB was completely mediated by MVPA, and MVPA was significantly associated with SB (Table 4). This means that the association between age at independent walking and SB in model 2 is caused by indirect effect through MVPA. Thus, it is plausible that the timing of onset of walking in infancy may influence the amount of MVPA in childhood, and then MVPA may cause the differences in sedentary time.

Independent walking, or upright and bipedal walking, a unique distinction of the human species, is the major motor developmental task during the first two years of life [33]. Age at onset of walking has often been used as an indicator of the progress of motor development in early life [14,17] and been shown to be associated with health risks in later life, such as bone strength [34] and blood pressure [35]. The age at which a child first walks independently varies widely from person to person and typically ranges from 8 months to 17–18 months, while 2.7% of children were not be able to walk independently at 24 months in a healthy sample [22]. Muscle strength and balance-control are thought to be rate-limiting factors for the onset of walking [33]; however, the timing of the onset of walking is modifiable. Exercise intervention such as stimulations to facilitate walking accelerate the development of walking [36]. Zelazo et al. (1972) showed that a few minutes of daily stepping practice with an upright posture over several weeks result in the onset of walking occurring 1.3–2.2 months earlier compared with control groups [37]. A more recent study has also shown that nutritional intervention improves gross motor development in small infants [38]. It is currently unclear whether these interventions to accelerate the gross motor development may have long-term benefits.

In the present study, the earlier age at onset of walking was associated with spending more time in MVPA in children (Table 3). This result is in line with previous studies showing that walking at an earlier age is associated with higher PA levels in infants and toddlers [17,18]. Although the detailed mechanisms by which the timing of onset of walking has an association with the amount of MVPA are not fully understood, some factors associated with child-rearing practices (e.g., encouragement to move by parents or caregivers) may carry over to the childhood years and therefore may increase MVPA. Another possibility is that potential motor proficiency may be associated with both the timing of the development of walking and the amount of MVPA. The link between higher motor proficiency and increased PA is widely known [39,40]. A few studies have shown a relation between earlier infant motor development and higher motor proficiency in later life [23,24,41]. Ridgway et al. (2009) reported that the age at which infants stand unaided and walking supported were associated with muscle strength, muscle endurance, and cardiorespiratory fitness at the age of 31 years [41]. Kuh et al. (2006) found that the age at which a child walked was a predictor of standing balance, chair rises [23], and muscle performance [24] at the age of 53 years. Prospective studies that include the assessment of factors associated with child-rearing practices and motor fitness would be beneficial to elucidate a causal association between the development of walking and PA patterns in later life.

In the case of analyzing by gender, the association between motor development and LPA was no longer significant in boys, while girls still showed a significant association. It is not clear why LPA was not predictive in boys, however, fewer sample size for boys would be partly related to these results and otherwise the patterns of LPA may be more strongly associated with other determinants such as psychological, social, and behavioral factors in boys.

The present study has several limitations. First, we relied on the parents’ recall for information on birth weight and the age at which the children walked independently. Registered birth weight has been shown to be in good agreement with birth weight recalled by mothers of school children age 8–11 years (interclass coefficient = 0.95, mean differences = 1.2 g) [42]. The reliability of age at walking recalled by parents of children six years of age and older is unknown [43]. As this variable is also based on parental reports rather than on an objective assessment, reporting bias may exist. In addition, this parameter is also accompanied by a problem of definition: independent walking has been variously defined as walking two or three steps without support [44] or walking at least five steps independently [45], or it was not specifically defined [34]. Therefore, recall bias, reporting bias, and the definition problem might potentially influence the data. Nevertheless, the 10th, 50th, and 90th percentile values for age at the onset of independent walking in this study (10.0, 12.0, and 15.0 mos., respectively) were almost the same as in the WHO Motor Development Study (10.0, 12.0, and 14.4 mos., respectively) [22] in that motor development was objectively assessed. Second, as this was a retrospective study, long term follow-up studies are needed to confirm the influence of development timing of onset of walking on PA and SB in later age. While this remains to be investigated, this study provides valuable information among infants with a later age at onset of independent walking.

## Conclusion

In summary, the present study showed that a later age at onset of walking in infancy was associated with prolonged sedentary time in childhood. However, when MVPA was introduced as a covariate, this association was completely mediated by MVPA. MVPA was significantly associated with SB. These results indicate that a later age at onset of independent walking may have a negative influence on MVPA, with an associated increase in sedentary time in children. This suggests that the timing at which a child walked for the first time in early life may have long-term implications for subsequent activity patterns. Our findings also suggest that appropriate interventions which focus on increasing MVPA and thereby reducing SB may be beneficial in infants who demonstrate a later age at onset of independent walking.

## Supporting information

S1 Table Partial correlation matrix controlled for months of age in boys and girls separately. * *P* < 0.05; ** *P* < 0.01; *** *P* < 0.001. SB, sedentary behavior; LPA, light physical activity; MVPA, moderate-to-vigorous physical activity.
(PDF)

S2 Table Multiple linear regression analyses with SB or PA as the dependent variable in boys and girls separately. Model 1: Adjusted for months of age, schools, and accelerometer wear time. Model 2: As Model 1 plus birth weight and current weight. *B*, unstandardized regression coefficient; *β*, standardized regression coefficient. SB, sedentary behavior; LPA, light physical activity; MVPA, moderate-to-vigorous physical activity.
(PDF)

S3 Table Multiple linear regression analysis with SB as the dependent variable in boys and girls separately. Adjusted for months of age, birth weight, current weight, schools, and accelerometer wear time. *B*, unstandardized regression coefficient; *β*, standardized regression coefficient. SB, sedentary behavior; MVPA, moderate-to-vigorous physical activity.
(PDF)
