# Supplementary material for: Association between age at onset of independent walking and objectively measured sedentary behavior is mediated by moderate-to-vigorous physical activity in primary school children
Source: PLoS One. 2018 Sep 18;13(9):e0204030. doi: 10.1371/journal.pone.0204030 (PMC6143251; doi:10.1371/journal.pone.0204030)
Supplement: S1 Table — * P < 0.05; ** P < 0.01; *** P < 0.001. SB, sedentary behavior; LPA, light physical activity; MVPA, moderate-to-vigorous physical activity. (PDF) [file pone.0204030.s001.pdf]

S1 Table

Variables	Age at independent walking (mos.)	Height (cm)	Weight (kg)	SB (min/day)	LPA (min/day)	MVPA (min/day)
**Boys**						
Birth weight (g)	-0.079	0.117	0.129	-0.051	0.025	0.139
Age at independent walking (mos.)		-0.047	-0.125	**0.161***	-0.062	**-0.230****
Height (cm)			**0.711*****	**0.161***	**-0.218****	-0.012
Weight (kg)				**0.164***	**-0.181***	-0.048
SB (min/day)					**-0.725*****	**-0.570*****
LPA (min/day)						**0.378*****
**Girls**						
Birth weight (g)	-0.131	**0.184****	0.124	-0.020	-0.008	-0.035
Age at independent walking (mos.)		-0.094	-0.096	**0.152***	-0.099	**-0.174***
Height (cm)			**0.753*****	0.059	-0.008	-0.085
Weight (kg)				**0.154***	-0.118	-0.104
SB (min/day)					**-0.726*****	**-0.583*****
LPA (min/day)						**0.320*****
