# Supplementary material for: Association between age at onset of independent walking and objectively measured sedentary behavior is mediated by moderate-to-vigorous physical activity in primary school children
Source: PLoS One. 2018 Sep 18;13(9):e0204030. doi: 10.1371/journal.pone.0204030 (PMC6143251; doi:10.1371/journal.pone.0204030)
Supplement: S2 Table — Model 1: Adjusted for months of age, schools, and accelerometer wear time. Model 2: As Model 1 plus birth weight and current weight. B, unstandardized regression coefficient; β, standardized regression coefficient. SB, sedentary behavior; LPA, light physical activity; MVPA, moderate-to-vigorous physical activity. (PDF) [file pone.0204030.s002.pdf]

S2 Table

	SB (min/day)			LPA (min/day)			MVPA (min/day)		
Independent variables	*B*	*β*	*P*	*B*	*β*	*P*	*B*	*β*	*P*
**Boys**									
**Model 1**									
Age at independent walking (mos.)	8.87	0.12	0.087	-4.07	-0.07	0.335	-4.80	-0.19	**0.007**
**Model 2**									
Age at independent walking (mos.)	10.79	0.14	**0.035**	-5.40	-0.09	0.199	-5.40	-0.21	**0.002**
**Girls**									
**Model 1**									
Age at independent walking (mos.)	12.54	0.15	**0.005**	-8.25	-0.12	**0.029**	-4.29	-0.18	**0.004**
**Model 2**									
Age at independent walking (mos.)	13.05	0.16	**0.004**	-8.55	-0.13	**0.024**	-4.51	-0.19	**0.002**
