# Supplementary material for: Association between age at onset of independent walking and objectively measured sedentary behavior is mediated by moderate-to-vigorous physical activity in primary school children
Source: PLoS One. 2018 Sep 18;13(9):e0204030. doi: 10.1371/journal.pone.0204030 (PMC6143251; doi:10.1371/journal.pone.0204030)
Supplement: S3 Table — Adjusted for months of age, birth weight, current weight, schools, and accelerometer wear time. B, unstandardized regression coefficient; β, standardized regression coefficient. SB, sedentary behavior; MVPA, moderate-to-vigorous physical activity. (PDF) [file pone.0204030.s003.pdf]

S3 Table

	SB (min/day)		
Independent variables	*B*	*β*	*P*
**Boys**			
Age at independent walking (mos.)	0.80	0.01	0.844
MVPA (min/day)	-1.85	-0.63	**<0.001**
**Girls**			
Age at independent walking (mos.)	5.01	0.06	0.176
MVPA (min/day)	-1.79	-0.51	**<0.001**
